# Craniometric parameters for the evaluation of platybasia and basilar invagination on magnetic resonance imaging: a reproducibility study

**DOI:** 10.1590/0100-3984.2019.0068

**Published:** 2020

**Authors:** Alexandre Tejo Pereira de Brito Silva, Lucas Tejo Pereira de Brito Silva, Alysson Emannuel Neves Rodrigues Vieira, Cibelle Ingrid Estevão de Melo, José Jailson Costa do Nascimento, Carlos Fernando de Mello Júnior, Selene Cordeiro Vasconcelos, Severino Aires de Araújo-Neto

**Affiliations:** 1 Universidade Federal da Paraíba (UFPB), João Pessoa, PB, Brazil.; 2 Universidade Federal de Pernambuco (UFPE), Recife, PE, Brazil.

**Keywords:** Cephalometry, Basilar invagination, Craniovertebral junction, Odontoid process/abnormalities, Magnetic resonance imaging, Reproducibility of results, Cefalometria, Invaginação basilar, Junção craniovertebral, Processo odontoide/anormalidades, Ressonância magnética, Reprodutibilidade de resultados

## Abstract

**Objective:**

The present study aims to perform a reproducibility study of the clivus-canal angle (CCA), Welcker’s basal angle (WBA), and the distance from the odontoid process to Chamberlain’s line (DOCL) on magnetic resonance imaging (MRI).

**Materials and Methods:**

Two medical students and two radiologists respectively evaluated 100 and 50 consecutive MRI scans of adult skulls, selected randomly. Each examiner, working independently and blinded to the previous results, performed readings for each patient on two different occasions. Measurements were performed in T1-weighted sequences acquired in the midsagittal plane. The levels of intraobserver reproducibility and interobserver agreement were evaluated by calculating the intraclass correlation coefficients (ICCs) and the corresponding 95% confidence intervals.

**Results:**

The mean values obtained by the examiners were 150º for the CCA, 130º for the WBA, and 2.5 mm for the DOCL. The ICC for interobserver agreement was 0.980, 0.935, and 0.967, for the CCA, WBA, and DOCL, respectively, for the students, compared with 0.977, 0.941, and 0.982, respectively, for the radiologists, and 0.980, 0.992, and 0.990, respectively, for all of the examiners together. In the analysis of intraobserver agreement, the ICC ranged from 0.929 to 0.959 for the CCA, from 0.918 to 0.964 for the WBA, and from 0.918 to 0.981 for the DOCL.

**Conclusion:**

The measurement of the CCA, WBA, and DOCL appears to show excellent intraobserver reproducibility and interobserver agreement on MRI.

## Abstract (pt)

**Objetivo:**

Realizar um estudo de reprodutibilidade do ângulo clivocanal (ACC), ângulo basal de Welcker (ABW) e distância do odontoide à linha de Chamberlain (DOLC) em ressonância magnética (RM).

**Materiais e Métodos:**

Quatro examinadores, dois graduandos de medicina e dois radiologistas, avaliaram, respectivamente, 100 e 50 indivíduos adultos submetidos a RM de crânio, consecutiva e aleatoriamente. Cada um realizou duas leituras para cada paciente em diferentes ocasiões, de forma cega e independente. As análises de concordância intraobservador e interobservador foram realizadas pelo coeficiente de correlação intraclasse (CCI), com intervalo de confiança de 95%.

**Resultados:**

As medidas médias, considerando todos os examinadores, foram: ACC = 150º, ABW = 130º, DOLC = 2,5 mm. A análise interobservador entre os estudantes revelou CCI de 0,980, 0,935 e 0,967 para ACC, ABW e DOLC, respectivamente, e para os radiologistas, CCI de 0,977, 0,941 e 0,982, respectivamente. A análise interobservador entre estudantes e radiologistas revelou CCI de 0,980, 0,992 e 0,990, respectivamente. Em relação à análise intraobservador, as medidas do ACC tiveram CCI variando entre 0,929 e 0,959, ABW entre 0,918 e 0,964 e DOLC entre 0,918 e 0,981.

**Conclusão:**

ACC, ABW e DOLC obtiveram excelentes reprodutibilidades intraobservador e interobservador na RM.

## INTRODUCTION

The craniovertebral junction (CVJ) marks the transition between the brain and the spinal cord; it is responsible for cranial mobility and stability. The CVJ region comprises several anatomical structures-including the occipital bone, the foramen magnum, the atlantoaxial joint, and ligaments, as well as major vascular and neural structures-and is subject to congenital or acquired conformational abnormalities such as platybasia and basilar invagination^(1-4)^. Platybasia refers to flattening of the skull base, whereas basilar invagination refers to projection of the odontoid process toward the posterior fossa^(4,5)^. The two alterations can occur in conjunction. Neurological symptoms of spinal cord and brainstem compression can arise in basilar invagination^(4,6)^. Basilar invagination can be classified as type A or type B. In type A, there is atlantoaxial instability, in which the dislocated odontoid process passes through the plane of the foramen magnum. In type B, the invagination of the base of the skull brings the upper cervical column into a more cephalic plane in relation to the occipital base. However, in type B, the odontoid process remains aligned with the anterior arch of the C1 vertebra and therefore does not invade the foramen magnum^(6)^. Apparently, each type stems from a different pathophysiological process, type A being predominantly a dislocation and type B, because of some degree of bone dysplasia, usually being occipital.

Anomalies of the CVJ are particularly common in India and in the northeastern region of Brazil^(7-9)^; it is notable that nearly all cases in northeastern Brazil are of type B, whereas nearly half of those in India are of type A. In both of those countries, the reported number of surgical cases is among the largest in the world^(10-12)^. Studies indicate that type B basilar invagination may be related to the flattened conformation of the calvaria, or brachycephaly, a phenotype found in approximately 80% of the population in northeastern Brazil, a prevalence much higher than that found in samples from western Europe and the North America^(10,13)^. Since 2013, Frade et al.^(11)^ have been using MRI to evaluate skull samples from northeastern Brazil. The authors found evidence that CVJ anomalies are more prevalent in the inland zone than in the coastal zone. In view of the epidemiological, pathophysiological, and (certainly) therapeutic peculiarities that distinguish the types of basilar invagination, the present study is restricted to the study of type B.

Diagnostic imaging based on the use of craniometric parameters, essentially composed of lines and angles, is crucial to the evaluation of CVJ abnormalities. The clivus-canal angle (CCA), Welcker’s basal angle (WBA), and the distance from the odontoid process to Chamberlain’s line (DOCL) are three of the most commonly used craniometric parameters^(2,4,5,14-16)^. These parameters were initially proposed for conventional X-ray examinations, the only diagnostic imaging tool available at the time^(14,15,17)^.

After computed tomography (CT) and magnetic resonance imaging (MRI), which is currently the gold standard, came to be available for clinical use, the old radiographic parameters for CVJ began to be applied in the new methods, as much for treatment as for research. The main advantage of MRI over CT is that it allows better visualization of neural structures, as well as of bone, although both methods avoid the superimposition of bone structures observed on X-rays^(2,17,18)^. However, validation studies of the use of traditional radiographic craniometric parameters such as CCA, WBA, and DOCL in MRI examinations, and of their respective reproducibility, are scarce in the literature^(11,14,17,19)^.

The objective of this study was to evaluate the reproducibility of the CCA, WBA, and DOCL in MRI. We also compared the performance of medical students and experienced radiologists in the craniometric analysis of CVJ.

## MATERIALS AND METHODS

This was a retrospective study for the validation of diagnostic tools using an imaging method different from what was initially used. The project was approved by the institutional research ethics committee.

### Study design and sample

Between 2016 and 2017, we studied the reproducibility of the CCA, WBA, and DOCL measurements in the MRI skull examinations of 100 adult patients. The examinations were originally performed between 2011 and 2012 at a private imaging clinic, subsequently being selected at random and consecutively. The examinations had been performed as outpatient procedures upon spontaneous request, for a variety of medical indications unrelated to our study protocol. Patients under 18 years of age were excluded. Other exclusion criteria were a patient history or imaging signs of skull base surgery and image quality that was insufficient to identify the anatomical landmarks needed in order to measure the parameters. Because the study objective was to determine the reproducibility of the MRI craniometry across its entire range of possible measurements, we did not exclude cases of platybasia or basilar invagination occasionally found in the MRI examinations. However, estimating their prevalence or specific pathological characteristics was not the focus of this study. For the reasons already given, patients with type A basilar invagination were not included.

### Imaging method

The MRI examinations were performed in a 0.35-T scanner (Magnetom; Siemens Medical Solutions, Erlangen, Germany). The measurements were mapped in a sagittal, three-dimensional, volumetric, isotropic magnetization-prepared rapid gradient-echo sequence, with a slice thickness of 0.9-1.1 mm, in the median slice that best demonstrated, concomitantly, the tip of the odontoid process and the mesencephalic aqueduct. To achieve a signal-to-noise ratio similar to what is normally obtained with a high-field device, the number of acquisitions in that sequence was increased and the acquisition time was therefore extended to approximately 9 min, comparable to the image resolution parameters applied in a 1.5-T MRI scanner with a 4-5 min acquisition time. The resulting images, in the Digital Imaging and Communication in Medicine format, were processed with OsiriX software, version 3.9.2 (Pixmeo SARL, Bernex, Switzerland).

### Craniometry

**Figure 1:**
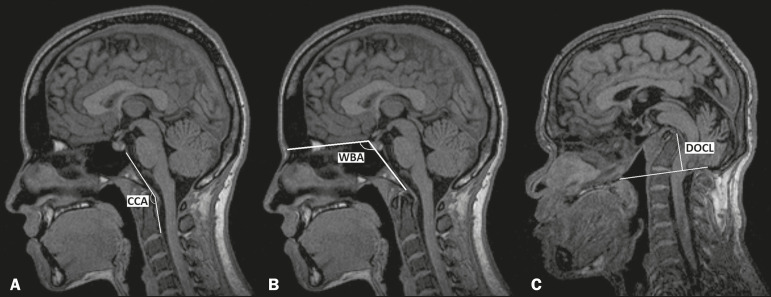
Midsagittal T1-weighted MRI. **A:** Measurement of the CCA. **B:** Measurement of the WBA. **C:** Measurement of the DOCL.

As described by Smoker et al.^(15)^, the CCA was defined as the angle between a line drawn along the posterior surface of the clivus and a line tangential to the posterior edge of the odontoid process and the body of the axis. The WBA was defined as the angle between a line running from the nasion to the tuberosity of the sella turcica and a line running from that point to the basion. Chamberlain’s line was drawn from the posterior edge of the foramen magnum (opisthion) to the dorsal edge of the hard palate; the DOCL was measured from the line perpendicular to Chamberlain’s line to the tip of the odontoid process^(20)^, as shown in Figure 1.

The examiners were divided into two groups: one composed of two 3rd-year medical students; and one composed of two radiologists, each with more than 15 years of experience in neuroimaging. The students received prior training for measuring craniometric parameters, according to the guidelines in the literature, evaluating, in a pilot study that preceded the present research protocol, 10 randomly chosen examinations that were not part of our sample. The students evaluated 100 MRI examinations, and the radiologists evaluated the first 50 examinations of that same sample, at which point the analysis reached statistical significance, which justified discontinuing the readings by the radiologists. Each examination was read twice, with a minimum of 3 weeks between measurements to mitigate any memory bias. The measurements were recorded on spreadsheets maintained by assistants unconnected to the reading procedure, so that the examiners were blinded to their previous results and the results of the other examiners.

### Statistical analysis

The statistical analysis was performed with the IBM SPSS Statistics software package, version 20.0 (IBM Corp., Armonk, NY, USA). The intraobserver reproducibility and interobserver agreement were assessed by calculating the intraclass correlation coefficient (ICC), interpreted in terms of the level of agreement, as follows^(21)^: poor (< 0.40); moderate (0.40-0.59); good (0.60-0.74); or excellent (> 0.74). The arithmetic mean of two readings from each examiner was used for the interobserver analysis. To compare the two groups (medical students and radiologists), we used the mean of the four measurements from each group. The comparison between genders was made with the Mann-Whitney U test. Spearman’s correlation coefficient was calculated to determine the relationship between the measurements and patient age. To illustrate interobserver agreement, we constructed Bland-Altman plots, in which each point refers to an examination and its location on the y-axis expresses the difference between the two measurements^(22)^.

## RESULTS

We evaluated the MRI examinations of 100 patients (55 females and 45 males). The mean age was 45.7 ± 18.1 years (range, 18-88 years). There were no significant gender-related differences for the craniometric parameters. In addition, age was not found to correlate significantly with any of the craniometric parameters (CCA: r = −0.094; *p* = 0.351; WBA: r = −0.065; *p* = 0.523; DOCL: r = 0.041, *p* = 0.684). Overall, the mean CCA was 150 ± 11º (range, 119-176º), the mean WBA was 130 ± 8.5º (range, 115-158º), and the mean DOCL was 2.5 ± 5.8 mm (range, -5.7 mm to 17.4 mm).

**Figure 2:**
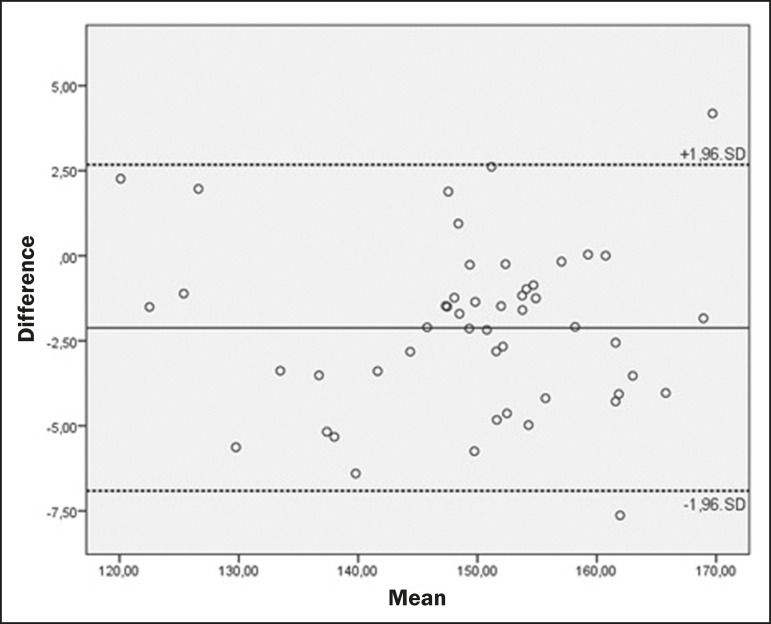
Bland-Altman plot of the interobserver agreement for the CCA measurements between the student and radiologist examiners, showing the difference between the mean values of each group (students − radiologists) with confidence intervals calculated by the formula mean difference ± 1.96 × standard deviation.

**Figure 3:**
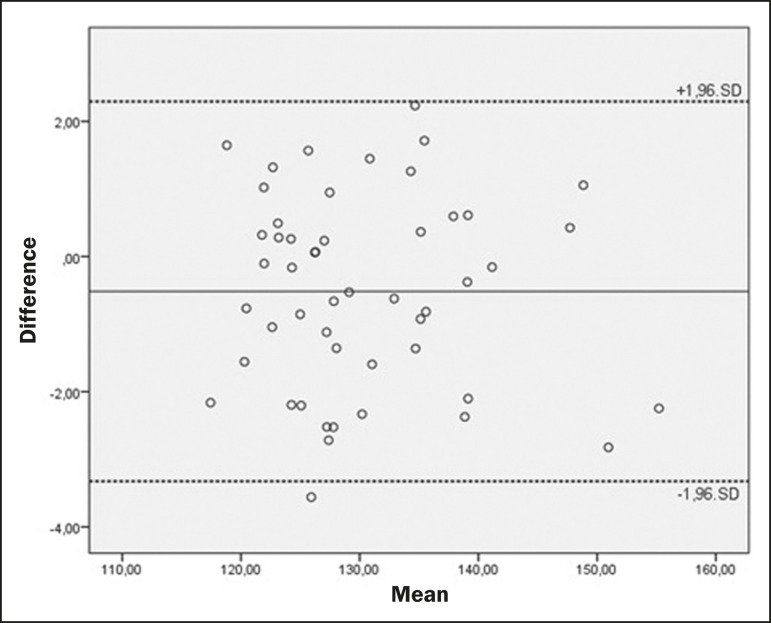
Bland-Altman plot of the interobserver agreement for the WBA measurements between the student and radiologist examiners, showing the difference between the mean values of each group (students − radiologists) with confidence intervals calculated by the formula mean difference ± 1.96 × standard deviation.

**Figure 4:**
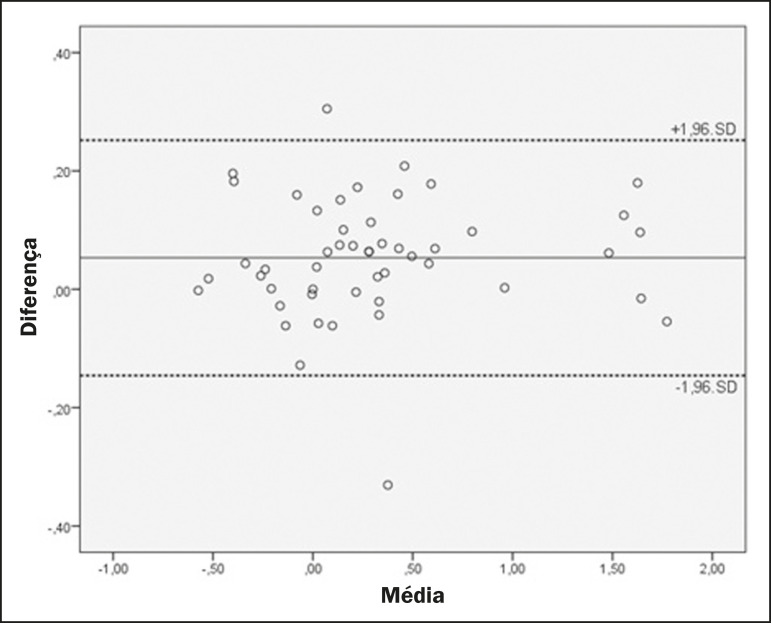
Bland-Altman plot of the interobserver agreement for the DOCL measurements between the student and radiologist examiners, showing the difference between the mean values of each group (students − radiologists) with confidence intervals calculated by the formula mean difference ± 1.96 × standard deviation. Values are shown in centimeters.

The mean values and the ICCs for intraobserver reproducibility and interobserver agreement are shown in Tables 1 and 2, respectively. Bland-Altman plots of interobserver agreement for the CCA, WBA, and DOCL measurements are shown in Figures 2, 3, and 4, respectively.

**Table 1:** Analysis of intraobserver reproducibility.

	CCA	WBA	DOCL
Examiner (examinations)	ICC (95% CI)	ICC (95% CI)	ICC (95% CI)
Student A (n = 50)	0.929 (0.897-0.952)	0.936 (0.905-0.956)	0.942 (0.914-0.960)
Student B (n = 50)	0.959 (0.939-0.972)	0.941 (0.913-0.960)	0.918 (0.880-0.944)
Radiologist A (n = 100)	0.944 (0.901-0.968)	0.964 (0.937-0.979)	0.981 (0.966-0.989)
Radiologist B (n = 100)	0.929 (0.878-0.959)	0.918 (0.860-0.953)	0.939 (0.894-0.965)

95% CI, 95% confidence interval.

**Table 2:** Analysis of interobserver agreement

	Patients	CCA	WBA	DOCL
Group of examiners	N	ICC (95% CI)	ICC (95% CI)	ICC (95% CI)
Students	100	0.980 (0.968-0.988)	0.935 (0.651-0.976)	0.967 (0.908-0.984)
Radiologists	50	0.977 (0.953-0.988)	0.977 (0.953-0.988)	0.982 (0.967-0.990)
Students and radiologists	50	0.980 (0.891-0.993)	0.992 (0.985-0.996)	0.990 (0.977-0.995)

95% CI, 95% confidence interval.

## DISCUSSION

Anomalies of the CVJ can have clinical and surgical implications due to the risk of bone compression at the bulbo-medullary junction, leading to motor disorders such as hemiparesis, sensory disturbances, and compressive disorders of the brainstem and some cranial nerves^(15-17,23)^. In addition, these alterations force the CVJ to remain in a position of excessive kyphosis, which can compromise the local vascularization and generate chronic musculoskeletal pain^(17,23)^. Imaging methods make a valuable contribution to the diagnosis of these disorders.

Many of the most popular craniometric parameters used in detecting CVJ anomalies were first proposed and tested several decades ago for use in conventional X-ray examinations, which predominantly represent bone structures. On X-rays, the absolute dimensions are dependent on the distance from the film to the object and from the object to the radiation source. The positioning of the patient is subject to angulation; typically only frontal and lateral views are available in X-ray examinations of the skull. In addition, bone reference points are sometimes difficult to visualize due to the superimposition of structures that were not the focus of the present study. On MRI examinations, the bone and nerve structures are well defined, the acquisition of cross-sectional images in multiple planes overcoming the disadvantage of superimposed structures on X-ray examinations^(2,17)^.

There have been many descriptive studies dealing with population profiles and limits of normality for craniometric parameters in CVJ anomalies, ranging from those evaluating X-rays in the first half of the twentieth century to those evaluating the new cross-sectional methods (CT and MRI) from the 1980s to the present. However, the interchangeability of numerous parameters, primarily those originally developed for use with X-rays, between the different imaging methods lacks the proper validation, with respect to reproducibility and accuracy, because of the differences in the physical principles of generating images, as discussed here. Therefore, scientific evidence of diagnostic validation, including measurements of reproducibility (precision) are needed in order to substantiate the safe application of these diagnostic parameters in the clinical routine.

Although imaging reports of CVJ anomalies are scarce, CT and X-ray reports are more numerous than are MRI reports. Xu et al.^(24)^ investigated the interobserver reliability and accuracy of measurement of the clivodens angle and the CCA, finding that they had a Kendall’s tau of 0.891 and 0.855, respectively, and that both showed good accuracy. Although the CCA presented the worst results, the area under the receiver operating characteristic curve (AUC) was 0.916 and the difference between the two measurements was not statistically significant.

Shoda et al.^(25)^ and Arponem et al.^(26)^ evaluated the reproducibility of measuring Chamberlain’s, McRae’s, and McGregor’s lines on lateral X-rays of the skull. Shoda et al.^(25)^ found that the ICCs for interobserver agreement were 0.939, 0.802, and 0.97, respectively. Arponem et al.^(26)^ sought to evaluate variations in the identification of different anatomical landmarks and their influence on the measurement of the parameters mentioned above, concluding that such variations resulted in only a small clinically significant difference. In a study assessing the craniometry findings of 100 asymptomatic patients by measuring 17 craniometric parameters extracted from CT examinations, including the three parameters evaluated in the present study, Batista et al.^(27)^ drew attention to the lack of studies testing the reproducibility of CT evaluation of craniometric parameters. The results obtained by those authors were merely cross-sectional and descriptive.

There have been only a few studies of the reproducibility of measuring CVJ craniometric parameters on MRI. In one of those studies, Wang et al.^(17)^ analyzed the intraobserver reproducibility and interobserver agreement for the measurement of the cervicomedullary angle, which also evaluates the risk of compression of the spinal cord. The cervicomedullary angle is described as the angle formed by the intersection of lines drawn parallel to the anterior surface of the spinal cord and the brain stem. In that study, the mean cervicomedullary angle was 158.46º and the measurements were highly reproducible. Martin et al.^(28)^ evaluated the intraobserver reproducibility and interobserver agreement for the measurement of the CCA and Grabb’s line on MRI, which were the targets of their study. Grabb’s line is used in the evaluation of patients with Chiari malformation and is described as the perpendicular distance between the basion and the bottom edge of the C2 vertebra. The authors employed four evaluators, including an undergraduate student, and observed high reproducibility between measurements, with ICCs of 0.879 for Grabb’s line and 0.916 for the CCA; they found no significant correlation between the length of clinical experience and the ICCs of the measurements. Nascimento et al.^(19)^ assessed the accuracy of five craniometric parameters including the CCA, WBA, and DOCL, while investigating type B basilar invagination. Those authors employed two examiners, evaluating intraobserver reproducibility and determined the AUC for each measurement. The diagnostic accuracy (AUC) was above 0.8 for all parameters, being higher for the DOCL and for Boogard’s angle. However, in that study, interobserver agreement was not analyzed and there were no student examiners.

Koenigsberg et al.^(14)^ made a descriptive comparison between the WBA values obtained in MRI examinations and those obtained in previous studies that used different measurement techniques by means of traditional X-ray. However, the agreement across studies was not measured statistically. In addition, the authors did not evaluate interobserver agreement or intraobserver reproducibility.

The parameters tested in the present study demonstrated extremely high intraobserver reproducibility and interobserver agreement. In all comparisons, the ICC was above 0.90, with narrow confidence intervals and a minimum above 0.8 (considered excellent), except for a single value (0.651) recorded in the interobserver comparison between the two student examiners. The Bland-Altman plots corroborated the high agreement between the measurements, with minimal mean differences. The performance of the medical students was comparable to that of the radiologists, which allows us to assume that years of experience are not a determinant of high accuracy in applying these diagnostic tests to MRI scans.

In the present study, if the limits of normality suggested by the literature were applied, 51 patients would be considered abnormal on the basis of their CCA (< 150º) and 9 would be considered abnormal on the basis of their WBA (> 140º). On the basis of their DOCL, one of the most widely used parameters, half of the patients would be diagnosed with basilar invagination if the limit of normality were set at 2 mm, as would 18 of them if that limit were set at 5 mm. For a population that underwent MRI examination of the skull for random and various clinical reasons, these are unexpectedly high rates of CVJ abnormalities, which leads us to assume that population characteristics specific to northeastern Brazil account for the fact that the distribution of values for these parameters differed from that reported in studies conducted in other countries.

The shape of the skull can vary among different populations, being dependent on ethnic, anthropological, and geographical factors^(10,11,13,29)^. For example, brachycephaly is much more prevalent in certain regions, such as northeastern Brazil and Southeast Asia^(7,9,13)^. Since the 1970s, surgeons in Brazil have reported cases in which CVJ abnormalities and brachycephaly occurred in conjunction^(12,29)^. It is possible that the morphology of the cranial vault and the shape of the skull base are influenced by the same constitutional factors, being flatter in Brazil than in Europe, for example. Therefore, there is a need for additional studies obtaining independent data and, if this trend is corroborated, proposing specific parameters for Brazil.

## CONCLUSION

The present study demonstrated that the reproducibility of CCA, WBA, and DOCL measurements on MRI is consistently high among trained medical students and among experienced radiologists. After basic training, medical students can contribute to craniometric measurements on MRI, without compromising the methodology or statistical analysis.
